# GLOBAL DIFFERENCES IN ADAPTATION TO SOCIETAL AGING: A CROSS-SECTIONAL COMPARISON OF 143 COUNTRIES

**DOI:** 10.1093/geroni/igac059.2129

**Published:** 2022-12-20

**Authors:** Cynthia Chen, Kenwin Maung, Jemima Koh, John W Rowe

**Affiliations:** National University of Singapore, Singapore, Singapore; University of Rochester, Rochester, New York, United States; National University of Singapore, Singapore, Singapore; Columbia University, New York City, New York, United States

## Abstract

Societal ageing is our success story of advancing public health and medicine in reducing the risk of premature death, the control and prevention of diseases, and improvement in social and economic development. Almost all countries are experiencing growth in size and proportion of older persons. In 2019, 703 million older persons were aged 65 and above, projected to double to 1.5 billion by 2050. We construct a multidimensional ageing Index that extends the Ageing Society Index, to account for global differences in societal ageing from 2015 to 2019 for 18 OECD countries. The Index is a weighted sum of scores for five domains that are important for societal ageing: well-being, productivity and engagement, equity, cohesion, and security. We compare the overall index and domain scores between countries. High-income countries dominated the top rankings. The overall Ageing Society Index ranged between 35.0 for Gabon in Africa to 74.6 for Switzerland in Europe. Top-performing countries were high-income countries from the European and Oceania regions, while the worst-performing countries were countries from the African region. Domain scores ranged from 19 (India) to 91.6 (Singapore) for well-being, 12.8 (Mozambique) to 88.5 (Finland) for equity, 32.8 (Gabon) to 88.8 (Uzbekistan) for cohesion, and 27.8 (Madagascar) to 88.8 (Switzerland) for security. Our multidimensional index helps identify specific societal gaps for policymakers to address. Furthermore, the cross-country comparison can be instructive for policymakers to adapt the experiences of successful countries to domestic policies.

